# A concurrent excitation and inhibition of dopaminergic subpopulations in response to nicotine

**DOI:** 10.1038/srep08184

**Published:** 2015-02-02

**Authors:** Raphaël Eddine, Sebastien Valverde, Stefania Tolu, Daniel Dautan, Audrey Hay, Carole Morel, Yihui Cui, Bertrand Lambolez, Laurent Venance, Fabio Marti, Philippe Faure

**Affiliations:** 1 Sorbonne Universités, Neuroscience Paris Seine, CNRS UMR 8246, INSERM U 1130, UPMC Univ Paris 06, UM119, 75005 Paris, France; 2 Dynamics and Pathophysiology of Neuronal Networks, Center for Interdisciplinary Research in Biology, INSERM U667, College de France, 75005 Paris, France

## Abstract

Midbrain dopamine (DA) neurons are key players in motivation and reward processing. Increased DA release is thought to be central in the initiation of drug addiction. Whereas dopamine neurons are generally considered to be activated by drugs such as nicotine, we report here that nicotine not only induces excitation of ventral tegmental area (VTA) DA cells but also induces inhibition of a subset of VTA DA neurons that are anatomically segregated in the medial part of the VTA. These opposite responses do not correlate with the inhibition and excitation induced by noxious stimuli. We show that this inhibition requires D2 receptor (D2-R) activation, suggesting that a dopaminergic release is involved in the mechanism. Our findings suggest a principle of concurrent excitation and inhibition of VTA DA cells in response to nicotine. It promotes unexplored roles for DA release in addiction contrasting with the classical views of reinforcement and motivation, and give rise to a new interpretation of the mode of operation of the reward system.

Midbrain dopamine (DA) neurons from the ventral tegmental area (VTA) are involved in several brain processes including reinforcement, associative learning, motivation, and are critically involved in the pathophysiology of addiction. Nicotine, the primary addictive component of tobacco, exerts its reinforcing effects by modifying VTA DA neurons activity through its action on nicotinic acetylcholine receptors (nAChRs)^1^,^2^,^3^,^4^ and through the subsequent DA release^5^ in target areas such as the Nucleus Accumbens (NAc) and the prefrontal cortex (PFC).

DA VTA cells can be segregated into different functional subpopulations, according to their spontaneous electrophysiological activity, protein expression, area of axonal projection^6^,^7^, and responses to appetitive and aversive stimuli^8^,^9^,^10^. While the vast majority of studies that have examined the interactions between DA VTA neurons and nicotine report a homogenous activation and an increase in dopamine release in target areas^3^,^5^,^11^,^12^,^13^,^14^, some results suggest that DA cells response to nicotine is more heterogeneous than previously thought^15^. For instance, nicotinic activation of posterior VTA DA neurons has been reported to be higher than in the anterior VTA^13^,^16^. More interestingly, in rare cases, some cells seem to show an inhibition of firing in response to nicotine^2^,^15^,^16^,^17^. However this effect has neither been characterized nor linked with physiological or anatomical data. Yet the study of such heterogeneous effects of nicotine at the level of VTA DA neurons seems crucial in order to understand the complex action of this drug and to dissect the mechanisms of the control of nicotine consumption.

Here, using in vivo single cell recordings in mice, we show that VTA DA neurons are divided in two subpopulations, displaying either an excitation or an inhibition following nicotine administration. We show that these subpopulations are electrophysiologically similar but anatomically distinct, being partially segregated along the mediolateral axis of the VTA. Furthermore, these subpopulations differ from those distinguished by their excitatory/inhibitory response to painful stimuli. At last, we show that inhibition, but not excitation, requires functional D2 receptor (D2-R). The mechanism of this medial inhibition suggests an anatomically dependent network effect relying on activation of D2Rs. These results provide insights into the study of the effects of nicotine and raise new questions concerning the instatement of nicotine addiction.

## Results

### A subpopulation of VTA DA neurons is inhibited by nicotine

**Figure 1:**
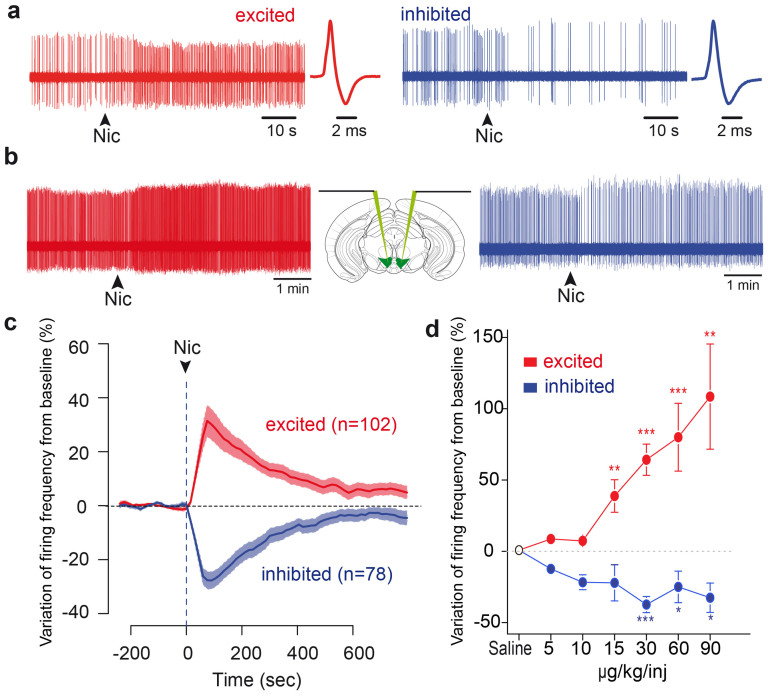
A subpopulation of VTA DA neurons is inhibited by nicotine. (a) Raw *in vivo* single cell extracellular recordings of an excited (left) and an inhibited VTA DA neuron (right) at the time of nicotine (30 μg/kg) intravenous injection (indicated by arrowheads). Corresponding wide action potentials, typical of DA neurons, are displayed on the right. (b) Schematic showing a mouse brain in which electrodes are lowered in both hemispheres. Raw extracellular simultaneous recordings of single DA neurons located in right and left VTA, at the time of nicotine (30 μg/kg) intravenous injection (arrowheads). This pair of neurons consists of an excited cell (red, left) and an inhibited cell (blue, right), showing that the responses to nicotine are not state dependent. (c) Mean responses of excited (red, n = 102) and inhibited (blue, n = 78) VTA DA neurons. Data, expressed as variation of firing frequency from baseline, are presented as mean (thick curves) ± SEM (light curves). Arrowhead represents the time of nicotine injection. (d) Mean responses of excited (red) and inhibited (blue) VTA DA neurons to increasing doses of nicotine (excited at 5 μg/kg: n = 8, 10 μg/kg: n = 8, 15 μg/kg: n = 10, 30 μg/kg: n = 50, 60 μg/kg: n = 18, 90 μg/kg: n = 10; inhibited at 5 μg/kg: n = 2, 10 μg/kg: n = 5, 15 μg/kg: n = 5, 30 μg/kg: n = 27, 60 μg/kg: n = 15, 90 μg/kg: n = 4). Similar results are obtained whether considering a single neuron's response at different doses or when considering the mean response of the populations. (* p < 0.05, ** p < 0.01, *** p < 0.001)

We performed in vivo single cell electrophysiological recordings on anaesthetized mice, and recorded the response to an intravenous nicotine injection (30 μg/kg) of a pool of putative DA neurons (n = 180) identified based on their electrophysiological characteristics (i.e. firing frequency and action potential width^18^). Of these putative DA neurons, 13 were pharmacologically and immunocytochemically confirmed DA neurons, 23 and 48 were respectively pharmacologically or immunocytochemically confirmed. Importantly, the dose of nicotine injected is physiologically relevant as it corresponds to the dose that is intravenously self-administered by mice^19^. We found a consistent amount of DA neurons that did not display the expected increase of firing frequency in response to nicotine, but instead displayed a drop in their firing activity following the injection (Fig. 1a). Of the 180 neurons that received nicotine, 102 (56%) were excited while 78 (44%) were inhibited. We first show that, except for the firing pattern of inhibited cells that seemed slightly more irregular than in excited one (Coefficient of Variation 0.46 ± 0.03 in excited cells, 0.56 ± 0.03 in inhibited cells, p = 0.038, t-test), the electrophysiological properties of the inhibited and excited DA subpopulations displayed no differences (see Fig. S1A). Furthermore, since DA cell activity is influenced by the stage of sleep and deepness of anaesthesia^20^, we verified that these types of responses to nicotine do not depend on such side parameters and recorded pairs of DA neurons by descending one electrode in each side of the brain (Fig. 1b). Among 3 pairs of VTA DA neurons, 1 consisted of two oppositely responding neurons (i.e. one excited and one simultaneously inhibited). Analogously, 1 out of the 3 pairs contained two neurons belonging to different classes of spontaneous activity (i.e. one regularly and one irregularly firing). This ensures that the opposite responses to nicotine coexist in the brain, as well as the heterogeneity of spontaneous DA activities. Moreover, inhibited and excited cells both displayed an important modification of firing frequency upon nicotine injection (mean response for excited neurons: + 37.52 ± 5.8 %, p = 2.2e-16; for inhibited neurons: −34.57 ± 3.1%, p = 2.1e-14, exact Wilcoxon signed rank test), and the peak of the effect occurred at the same time in both populations (92.1 ± 3.5 seconds for excited and 95.0 ± 4.4 seconds for inhibited neurons, p > 0.05, asymptotic Wilcoxon rank sum test, Fig. 1c). Finally, the amplitude of the excitatory and inhibitory responses of DA neurons to nicotine is dose-dependent (Fig. 1d). Inhibition is thus a property of individual neurons and is not observed in excited neurons for any of the doses of nicotine studied.

### Inhibited and excited neurons are anatomically segregated within the VTA

**Figure 2:**
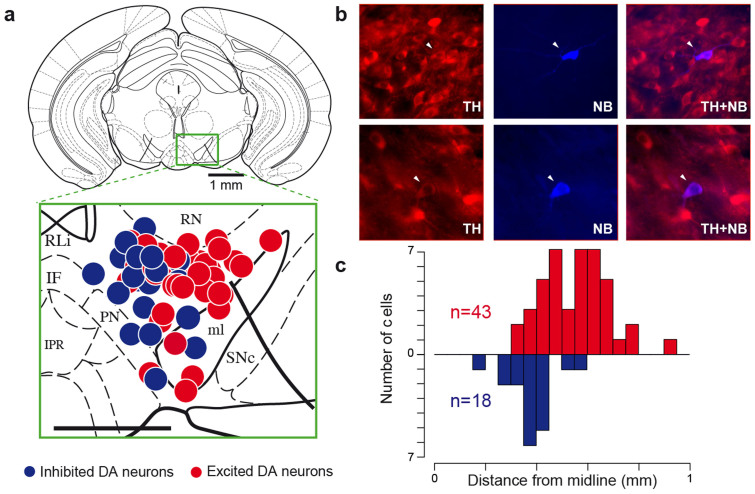
Inhibited DA neurons are located in the medial part of the VTA. (a) Coronal diagram at −3.52 mm from bregma onto which are positioned TH-positive cells labelled *in vivo*. Red and blue dots respectively represent excited (n = 43) and inhibited (n = 18) cells. Abbreviations: IF, interfascicular nu; IPR, interpedunc nu; ml, medial lemniscus; PN, paranigral nu; Rli, rostral linear nu; RN, red nu; SNc, substantia nigra compacta VTA, ventral tegmental area (scale bar: 0,5 mm). (b) Photomicrographs of two representative TH-positive (left) andneurobiotin-stained (middle) VTA DA neurons, one excited by nicotine (top), and the other inhibited (bottom). A merge micrograph (right) validates the DA phenotype of the recorded neuron. White arrow indicates labelled neuron. (c) Histogram of the labelled cells' anatomical distribution along the medio-lateral axis from the midline, inhibited neurons located at 378 ± 29 μm and excited neurons at 550 ± 32 μm, p < 0.001, Wilcox test.

Recently, subpopulations of DA neurons have been characterized according to their different properties such as anatomical localization, and D2-R expression^6^,^7^. We sought to determine whether the inhibited and excited DA subpopulations could similarly be discerned. 61 neurons (n = 43 excited cells and n = 18 inhibited cells, their respective response to nicotine is shown in in Fig. S1B) were labelled with the juxtacellular neurobiotin labelling technique^21^ within the VTA (Fig. 2a–b). We positioned each cell on coronal diagrams and quantified the anatomical distributions along the three stereotaxic axes: antero-posterior, medio-lateral, dorso-ventral. For illustration (Fig 2a), all labelled neurons (n = 61) were superposed onto a single Paxinos atlas schematic (AP −3.52), independently of their antero-posterior position (from 3000 to 4000 μm, Fig. S2). All of them were located in the PBP except 5 in the border of the ML and 1 in the PN. We found that inhibited neurons were located medially while excited neurons were located laterally within the VTA (Fig. 2c). These inhibited cells are mainly located in the medial PBP, but none of them were localized in the most medial part of the VTA (eg RLi, IF or even the medial part of the medial PBP). There was no difference along dorso-ventral, and antero-posterior axes (Fig. S2). Given that we were actively probing the medial region of the VTA, it is important to point out that the proportion of excited and inhibited DA cells reported here shouldn't be regarded as representative of the VTA layout.

### Responses to aversive events and to nicotine are not similarly distributed

**Figure 3:**
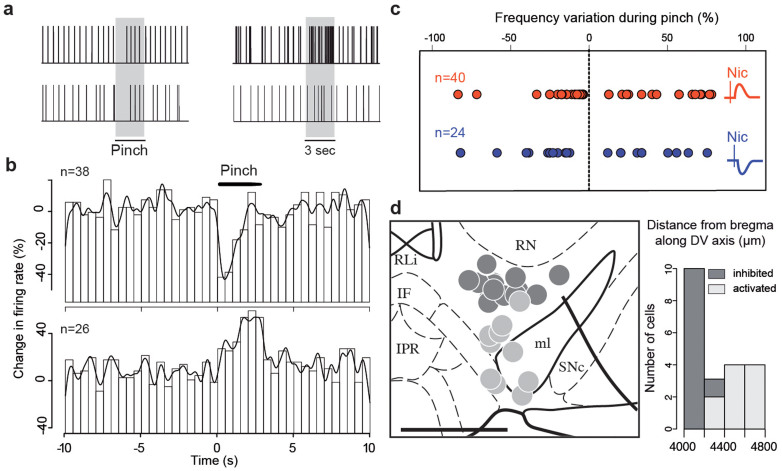
Inhibition and excitation of DA neurons in response to noxious stimuli. (a) Rasters representing the *in vivo* activity of two example neurons inhibited by a pinch (left) and two example neurons activated by a pinch (right). Grey area represents duration of pinch. (b) Population peristimulus time histogram (500 ms bins) averaging the activity of 38 pinch-inhibited neurons (top) and pinch-activated neurons (bottom, n = 26). (c) Frequency variation from baseline during pinch of neurons represented according to their response to nicotine. The responses of neurons excited by nicotine (red, n = 40) and inhibited by nicotine (blue, n = 24) do not correlate to their responses to a pinch. (d) Anatomical localizations within the VTA of confirmed DA neurons inhibited by a pinch (dark, n = 13) and activated by a pinch (light, n = 10), Activated neurons are located more ventrally (4515 ± 59 μm) than inhibited neurons (4230 ± 10 μm), p < 0.001, Wilcox test (scale bar: 0,5 mm).

Nicotine can act as both a rewarding and aversive stimulus^22^. Intriguingly, VTA DA neurons can also be either excited or inhibited by aversive events depending on their anatomical location^8^. To investigate a correlation between the responses to nicotine and those to an aversive stimulus, we delivered pinches during the recording of DA neurons that had been exposed to a nicotine injection (n = 64). We confirmed that dopaminergic neurons can either be excited (n = 24) or inhibited (n = 40) by pinches (Fig. 3a–b). However, we did not observe a correlation between DA neuron responses to nicotine and the responses to a pinch. Indeed, neurons that were excited or inhibited by nicotine could either be excited or inhibited by a pinch (Fig. 3c). Moreover, some of these DA neurons were labelled (n = 23) so as to confirm their DAergic identity and observe their anatomical localizations within the VTA. DA neurons inhibited by a pinch are located in the dorsal VTA (n = 13) and activated ones in the ventral VTA (n = 10) (Fig 3d), a result that confirms previous studies^8^. No differences were observed along the other axis which supports the fact that neuronal populations inhibited/activated by aversive stimuli do not match the populations inhibited/activated by nicotine, and therefore probably do not transmit the same motivational message.

### Mechanisms of the inhibition

**Figure 4:**
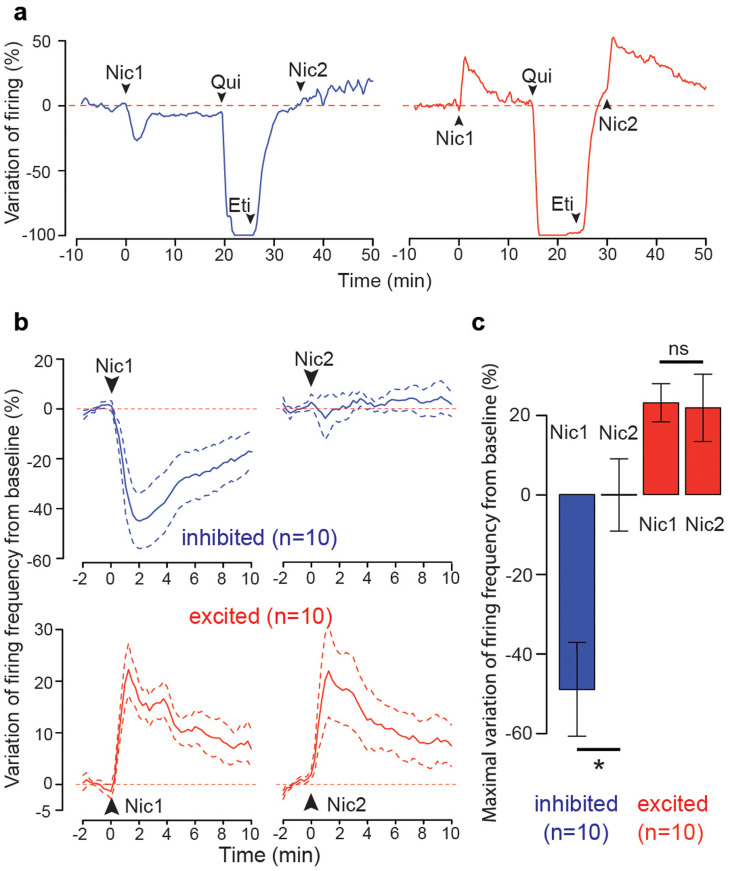
Abolition of nicotine inhibition but not excitation following D2-R blockade. (a) Example responses of an inhibited (left) and an excited (right) VTA DA neuron. Nic1, Nic2, Qui and Eti labelled arrowheads respectively represent the times of the first nicotine, second nicotine (both at 30 μg/kg), quinpirole (1 mg/kg), and eticlopride (1 mg/kg) intravenous injections. Red dotted lines represent baseline activity levels. (b) Mean responses to nicotine injection before (Nic1, left) and after (Nic2, right) D2 receptor (D2-R) blockade, in inhibited (top, n = 10) and excited (bottom, n = 10) DA neurons. Data are presented as mean (thick curves) ± SEM (dotted curves). Red dotted lines represent baseline activity levels. (c) Comparison of the maximal variation of firing frequency after nicotine injection before (Nic1) and after (Nic2) D2-R blockade, in inhibited (left, blue, n = 10) and excited (right, red, n = 10) DA neurons. For inhibited neurons, mean response before blockade: −44.8 ± 11.1 %, p < 0.01, exact Wilcoxon signed rank test; and after blockade: −2.4 ± 7.2 %, p > 0.05, exact Wilcoxon signed rank test; difference between the maximal amplitude of the two responses: 49.25 ± 16.5 %, p < 0.01, exact Wilcoxon rank sum test. For activated neurons, mean response before blockade: 20.5 ± 4.9%, p < 0.01, exact Wilcoxon signed rank test; and after: 20.2 ± 6.4 %, p > 0.05, exact Wilcoxon signed rank test; difference between the maximal amplitude of the two responses: −1.3 ± 8.6 %, p > 0.05, exact Wilcoxon rank sum test. Bars represent mean ± SEM; *: p < 0.01; ns: p > 0.05.

nAChRs are cation permeable, ligand-gated ion channels whose opening by nicotine induces membrane depolarization. Thus, while an increase in neuronal firing is the direct consequence of nAChR activation at the level of VTA DA neurons^3^,^15^, an inhibition of neuronal firing can only be indirectly explained by a yet-unidentified mechanism counteracting the direct excitation. The relative intensity of direct excitation versus indirect inhibition will then tilt the balance towards increased or decreased firing. To dissect the mechanisms of nicotine-induced inhibition, we first carried out an in vitro electrophysiological characterisation of nAChR evoked current in VTA DA neurons. We measured the currents induced by the nicotinic agonist DMPP in multiple neurons along the medio-lateral axis, but did not observe any difference in current amplitude based on the anatomical position of DA cells (Fig. S3). Inhibition thus seems to require an alternative mechanism. Activation of VTA DA neurons triggers dopamine release within the VTA, which exerts a negative feedback via the activation of D2 autoreceptors (D2-Rs)^23^. Thus nicotine-induced inhibition could rely on D2-R activation following somatodendritic DA release from excited VTA DA neurons. To test this hypothesis, we measured, *in-vivo*, the amplitude of the response in inhibited and excited cells after D2-R blockade. After a return to baseline activity from the first nicotine administration, we intravenously injected the D2-R family agonist quinpirole (1 mg/kg), which elicited a strong inhibition and allowed to assess the presence of D2-Rs on the cell (Fig. S4). We then intravenously injected the D2-R antagonist eticlopride (1 mg/kg), which reversed the quinpirole-induced inhibition but blocked further activation of D2-Rs, and subsequently performed a second nicotine injection (Fig. 4a).

Blockade of D2-R abolished the inhibition following nicotine administration (Fig. 4b–c) but did not change the nicotine-induced excitation. Thus the observed nicotine-induced inhibition, but not excitation, is mediated by D2-Rs, indicating that the underlying mechanisms involved in the regional differences of responses to nicotine are certainly linked to a balance between the nicotinic activation of DA neurons and the inhibition induced by D2-R activation.

## Discussion

The present work shows that a subpopulation of VTA DA neurons is inhibited by nicotine *in vivo*. This subpopulation cannot be distinguished from excited neurons by examination of spontaneous electrophysiological activity, including firing frequency, burst firing, coefficient of variation of the firing, and action potential shape (see Fig. S1A legend). However, inhibited cells are anatomically distributed in the medial part of the VTA, without segregation in the antero-posterior and dorso-ventral axes. We identify the mechanism of this inhibition as being dependant on an activation of D2-Rs.

In addition to DA neurons, the VTA contains about 40% of non-DAergic neurons^24^. These are mainly GABAergic but an additional subpopulation of glutamatergic neurons as well as mixed DA and glutamate co-releasing neurons have been identified in the medial part of the VTA^25^,^26^, which emphasizes the importance of precise identification of recorded neurons^27^,^28^. We identified DA neurons based on their location as well as on the set of unique electrophysiological and pharmacological properties that characterize these cells in vivo (see method). Overall, 61 cells with standard DAergic electrophysiological criteria have been labelled by neurobiotin. Most of them have been recorded in the PBP, the inhibited ones being localized in the medial PBP. Crucially, TH immunolabeling is a recognized gold-standard for identifying DA neurons in the VTA^27^,^28^. However, recent studies suggest that some TH positive neurons could also release glutamate or GABA, but this population is localized, in rats, in a more central zone initiated at the boundary between PBP and RLi^29^,^30^,^31^. In our study, electrophysiological criteria have always been validated by labeling and pharmacology (i.e we never found TH+ labelled cells with non-DA electrophysiological criteria), meaning that the risk that a putative DA cell is not dopaminergic is very low, even if we cannot exclude that some of these neurons co-express VGluT2. In addition, while nearly 80% of the TH+ neurons co-express D2-R in the lateral PBP in rats^31^, only 35 % of them co-express D2-R in the lateral part of the medial PBP. It is noteworthy that in the present study we have not identified medially located VTA DA neurons that do not respond to D2R pharmacology, as was previously shown *in vitro*^6^. Indeed, out of 18 neurons inhibited by nicotine and TH+ labelled in the medial VTA, 5 of them have been exposed to quinpirole, all of which were inhibited. However we cannot exclude that some of the recorded neurons lack D2-R. Questions could arise as to why this population of VTA DA neurons inhibited by nicotine hasn't been characterized before. The simplest explanation is that the superior sagittal sinus is located directly above this population, making it difficult to reach and record from these neurons.

Three mechanisms can be hypothesized to explain the inhibition of VTA DA cells: i) excitation of inhibitory GABAergic neurons; ii) D2-R activation following somatodendritic release of dopamine; iii) a network effect involving an inhibitory feedback loop coming from DA neuron projection areas. In previous studies, the cellular and behavioral effects of nicotine were shown to critically rely on the expression of the high affinity β2 nAChR in the VTA^3^,^15^,^32^. Indeed, in β2-/- KO mice, VTA DA responses to nicotine as well as sensitivity to nicotine-mediated rewards are abolished. However, re-expressing the high affinity β2 nAChR subunit specifically in VTA DA neurons partially restores these effects, but more compellingly inhibitory responses to nicotine are restored as well^32^. This strongly suggests that nicotinic activation of GABAergic interneurons of the VTA is not implicated in the nicotine-induced inhibition of DA neurons (but doesn't exclude that a GABAergic feedback from the NAc or elsewhere might be implied) and further supports the notion that the inhibition is a direct consequence of the excitation. In contrast, we have shown that this inhibition is mediated by D2Rs, which therefore implicates either an intra-VTA somatodendritic release of dopamine by neurons activated by nicotine, or a network effect with inhibitory feedback. Several studies report D2R-mediated IPSCs in VTA DA cells following stimulation^23^,^33^,^34^. The time scale of the inhibition described in these studies shows that the interval between excitation of the lateral DA cells and inhibition of the medial VTA cannot be distinguished with the temporal resolution achieved with our methods, which explains why both responses seem to be simultaneous in our experiments.

Our *in vitro* results suggest that the inhibitory effect of nicotine is a network-dependent outcome. A simple explanation would be the activation of a specific inhibitory feedback to medial VTA cells, but to our knowledge no evidence lends credence to this theory. However, differences in D2-R and DAT expression have been suggested. We show that the firing of some medial and lateral VTA neurons is similarly suppressed by D2-R agonists, suggesting that variations of D2-R expression might not explain our results. This could reflect the fact that dopamine reuptake by DAT varies along the mediolateral axis of the VTA^6^,^31^,^35^. Reduced DAT expression in the medial VTA could consequently result in reduced DA reuptake and in a prolonged rise of extracellular DA. Thus, nicotinic receptor mediated excitation is counterbalanced by a stronger D2R-mediated inhibition.

These data are consistent with previous studies suggesting that different subpopulations of the DA system have distinct functional roles. Such considerations often implicate the mesocortical versus the mesolimbic pathways. VTA DA neurons projecting to the PFC are located more medially in the VTA, while neurons projecting to the NAc were distributed within all the VTA. Furthermore, it has been proposed that D2R-mediated inhibition is absent in PFC- projecting neurons of rats and mice^6^,^36^,^37^, but this has been challenged by the observations that most prefrontal cortex-projecting neurons are sensitive to quinpirole^38^ in rats. Some of our inhibited neurons are inhibited by D2 agonist. We have as of yet no evidence for a specific projection of the inhibited versus the excited neurons. However, our results suggest that nicotine exerts its complex effects by sending opposing signals to different (sub-) regions of the brain. Whereas the functional significance of the excitation of the mesolimbic and cortical circuit has thoroughly been investigated and clearly plays an important role in reinforcement, the significance of a reduction of DA release in these areas has yet to be addressed. In particular, elevation of extracellular DA in the PFC has been linked to stimuli of negative motivational valence leading to aversion or anxiety^39^. Therefore it could be presumed that a decrease of dopamine release in the PFC results in motivationally positive phenotypes such as reduced anxiety. This would be consistent with the fact that smokers consider nicotine as a therapeutic strategy for relief of negative affect and anxiety. Moreover, such a decrease of dopamine could help promote impulsive behavior by disrupting inhibitory control over limbic regions.

Overall, the relative contributions of the different VTA dopaminergic pathways to the instatement of addiction still remain elusive, but our results show that in contrast to what has been previously expected, VTA DA neurons do not respond uniformly to nicotine. We suggest that the balance of inhibition and excitation of VTA DA neurons can be extended to other drugs and is a common mechanism underlying the initiation of addiction processes. These results further emphasize the complexity and diversity of the VTA, and demonstrate that the response to nicotine is most certainly built onto this diversity.

## Methods

Full Methods are available in the Supplementary Information.

### Animals

Experiments were performed on adult C57BL/6 male mice aged 8–21 weeks and weighing 25–35 grams. Animal care and experiments were conducted in accordance with European Ethical Committee guidelines and approved by the Charles Darwin Animal Experimentation Ethical Committee.

### In vivo electrophysiological recordings of VTA DA neurons

Single-unit extracellular recordings of VTA DA cells were performed in anesthetised mice as described in SI. Nicotine and alcohol were injected intravenously. DA cell identification is based on cumulative evidence principle: 1) electrophysiology criteria (see above) 2) quinpirole effect (see below) and 3) TH labelling. None of these criteria alone is sufficient for DA cell identification. However at least two of these criteria greatly decrease the probability of a false identification of a DA cell. The group of identified DA neurons comprised immunocytochemically labelled TH positive DA neurons (n = 61), and putative DA neurons inhibited by quinpirole (n = 23). Neurons that were labelled out of the VTA, or unlabelled neurons that did not inhibit in response to quinpirole were excluded. Pinch responses were measured as the change in firing rate from a 10 sec baseline before the pinch compared to 5 sec following the onset of the 3 sec tail-pinch.

### Immunocytochemical identification of recorded neurons and anatomical positioning

A series of juxtacellular labelling with neurobiotin and post-hoc TH immunocytochemistry allowed us to identify our cells as dopaminergic and to localize them in the VTA (see SI for description of the labelling method).

### Pharmacological experiments

Quinpirole hydrochloride, eticlopride hydrochloride, and gabazine (SR 95531 hydrobromide) were purchased from Tocris Bioscience. (-)-Nicotine hydrogen tartrate salt was purchased from Sigma-Aldrich. 15 min after the first nicotine injection, quinpirole 1 mg/kg was administered intravenously. 5 minutes later, eticlopride 1 mg/kg was administered intravenously. When the neuron activity was returned to baseline, a second nicotine injection was performed. Once D2-R pharmacology was applied, no further neurons were recorded and the animal was discarded.

### Statistical analysis

#### Measurement of neuronal activity

The analyses were done using the R software. Activity was quantified by overlapping 60 second windows shifted every 15 seconds. Percentage of spikes within bursts (%SWB) corresponds to the percentage of spikes discharged within a burst in a given time interval. Firing frequency response was quantified as a percentage of variation from baseline and %SWB response was quantified as a difference of percentage from baseline.

#### Subpopulations in response to nicotine

Subpopulations were automatically classified using variation of firing frequency and the following routine: considering the maximal variation from a short baseline (3 minutes prior to injection), within 3 minutes after injection, neurons displaying an increase in frequency were defined as “excited”, neurons displaying a decrease were defined as “inhibited”. Only very few neurons exhibit no clear modification of their firing rate after nicotine injection (i.e less than 5% of variation from baseline). We do not consider them as a specific subpopulation. Neurons (n = 10) displaying no significant variation from baseline (i.e maximal variation from baseline <5%) were pooled with the group of activated neurons.

#### Quantification of responses to nicotine

To evaluate the effect of nicotine each cell activity was rescaled to its baseline value. Spontaneous activity of the neurons was recorded for periods of a least 15 minutes to evaluate its stability. Only neurons with a stable activity (no sudden change in the baseline firing) were exposed to nicotine. For the quantification of the response to nicotine, the mean of 3 minutes of activity just prior to injection was taken as the value of baseline activity. For excited (respectively inhibited) neurons, the maximal (respectively minimal) value of the activity was measured within the baseline period (3 minutes) and within the response period (3 minutes). These two sets of values did not display normal distributions, and were then compared with paired non-parametric Wilcoxon signed-rank test. The peak of response to nicotine occurred within 2 minutes, and was thus correctly assessed by the use of 3 minutes periods.

#### Quantification of responses to D2-R pharmacology

Response to quinpirole was quantified in a manner analogous to nicotine response. Response to quinpirole occurred within tens of seconds. Effect of eticlopride developed slower, and was quantified as the mean activity during the 3 minutes preceding the second nicotine injection. This period was compared to a 3-minute baseline period preceding quinpirole injection. This allowed assessing the effects of eticlopride on the baseline activity of the neuron. The pre-quinpirole and post-eticlopride periods activities being normal, they were compared with a paired Student's t-test.

#### Anatomical segregation

For both populations of neurons (excited and inhibited), normality of the distribution of positions was assessed with a Shapiro-Wilk normality test. Normal distributions were compared with a Student's t-test, and non-normal distributions with a Wilcoxon test.

#### Total count of cells

In the paper, a total number of 276 neurons have been recorded. 180 neurons are used in Figure 1c and 77 neurons (no overlap with Fig 1c) for the figure 1d. Among these 180 neurons: 61 neurons are TH+ and used in the figure 2; 20 in the Figure 4. In the figure 3, 64 neurons are used, 45 of them have been used in the Fig 1c. Overall 36 neurons have received D2-R pharmacology. Again, once D2-R pharmacology was applied, no further neurons were recorded and the animal was discarded. A total of 1 to 4 neurons where generally recorded per mice.

## Author Contributions

R.E. and S.V. contributed equally to the work, having designed and performed the experiments, collected the data and written the paper. S.T., D.D., C.M. and F.M. participated in collecting and analysing the data. A.H., B.L., Y.C. and L.V. prepared the in vitro experiments. P.F. designed the experiments, analysed data and wrote the paper. All authors reviewed the manuscript.

## Supplementary Material

Supplementary Information supplementary information
