# Supplementary material for: A concurrent excitation and inhibition of dopaminergic subpopulations in response to nicotine
Source: Sci Rep. 2015 Feb 2;5:8184. doi: 10.1038/srep08184 (PMC4313096; doi:10.1038/srep08184)
Supplement: Supplementary Information [file srep08184-s1.pdf]

**A concurrent excitation and inhibition of dopaminergic subpopulations in response to nicotine**

Raphaël Eddine *PhD*[1†], Sebastien Valverde *MSc*[1†], Stefania Tolu *PhD*[1], Daniel Dautan *MSc*[1], Audrey Hay *MSc*[1], Carole Morel *PhD*[1], Yihui Cui *PhD*[2], Bertrand Lambolez *PhD*[1], Laurent Venance *PhD*[2], Fabio Marti *PhD*[1], Philippe Faure *PhD*[1*]

**Supplementary Information – Methods**

**In vivo electrophysiological recordings of VTA DA neurons**

8-12 weeks old male C57/Bl6 mice were deeply anaesthetized with chloral hydrate (8%), 400 mg/kg i.p., supplemented as required to maintain optimal anesthesia throughout the experiment. Glass electrodes containing 1.5% neurobiotin in 0.5% sodium acetate were lowered in the VTA according to stereotaxic coordinates derived from mouse brain atlas, and corrected empirically (antero-posterior : -3 to -4 mm ; medio-lateral : 0.1 to 0.7 mm ; dorso-ventral : -4 to -4,8 mm from bregma). To distinguish DA from non-DA neurons the following parameters were used: 1) firing rate (between 1 and 10 Hz); 2) action potential duration between the beginning and the negative trough superior to 1.1 ms. After a baseline recording of 5 minutes, a 10µl saline solution (0.9% sodium chloride) was injected into the saphenous vein, and after another 5 minutes, injection of 0.5mM nicotine tartrate (Sigma, USA, 30µg/kg concentration of nicotine base in a final volume of 10µl) was administered via the same route. Previous studies showed that nicotine can be intravenously self-administered at this dose in mice (19).

**Immunocytochemical identification of recorded neurons and anatomical positioning**

Neurons were electroporated and neurobiotin was expulsed from the electrode using positive current pulses (21). The mouse was then killed and the brain post-fixed in 4% paraformaldehyde. 60µm slices were cut on a vibratome. Post-hoc TH and neurobiotin immunocytochemistry allowed us to identify our cells as dopaminergic and to localize them in the VTA. Photomicrographs were taken to allow precise anatomical positioning of the labelled neurons. Examination of coronal slice shape and dopaminergic nuclei aspect, and comparison with serial coronal slices and Paxinos atlas schematics allowed to identify the antero-posterior level. The labelled neuron was then positioned as a dot on the schematic. Dorso-ventral and medio-lateral coordinates were precisely measured on the schematic according to the atlas benchmarks.

**Whole cell recordings**

8-12 weeks old male C57/Bl6 mice were deeply anesthesized with ketamine/xylazine. Coronal midbrain sections (250µm) were sliced using a vibratome (VT1000S; Leica) after intracardial perfusion of cold (4°C) artificial cerebrospinal fluid (ACSF) containing (in mM): 126 NaCl, 2.5 KCl, 1.25 NaH2PO4, 6 MgCl2, 26 NaHCO3, 25 sucrose (for osmolarity adjustment), 2.5 Glucose, 1 Kynurenate (pH 7.2, 325 mosM). After 1h at 37°C for recovery, individual slices were transferred to a recording chamber and continuously superfused at 2 ml/min with oxygenated ACSF containing (in mM) : 126 NaCl, 2.5 KCl, 1.25 $NaH_2PO_4$, 2 $CaCl_2$, 1 $MgCl_2$, 26 $NaHCO_3$, 15 sucrose, 10 Glucose (pH 7.2, 325 mosM) at room temperature (for longer conservation). Patch pipettes (3–6 MΩ) were pulled from borosilicate glass (Harvard Apparatus LTD, Kent, UK) on a micropipette puller (Model PP-83, Narishige, Tokyo, Japan) and filled with 8

μl of internal solution containing (in mM): 144 K-gluconate, 3 $MgCl_2$ , 10 HEPES, 0.5 EGTA and 3 mg/mL biocytin.). Whole-cell recordings were performed using a patch-clamp amplifier (Multiclamp 700B, Molecular Devices, Sunnyvale, CA) connected to a Digidata 1440A interface board (Molecular Devices). Signals were amplified and collected using the data-acquisition software pClamp 10.2 (Molecular Devices). DMPP (100μM, sigma) was applied through a local perfusion system in the presence of a cocktail of antagonists: 6-Cyano-7-nitroquinoxaline-2,3-dione (CNQX, 10μM), 2-amino-5-phosphonovalerate (APV, 50μM), Gabazine (GBZ, 1μM), , haloperidol (2μM), atropine (8μM) from Sigma (Saint Louis, MO) and CGP55845 (4μM) from Tocris (Bristol, GB).

**Supplementary Information – Figures**

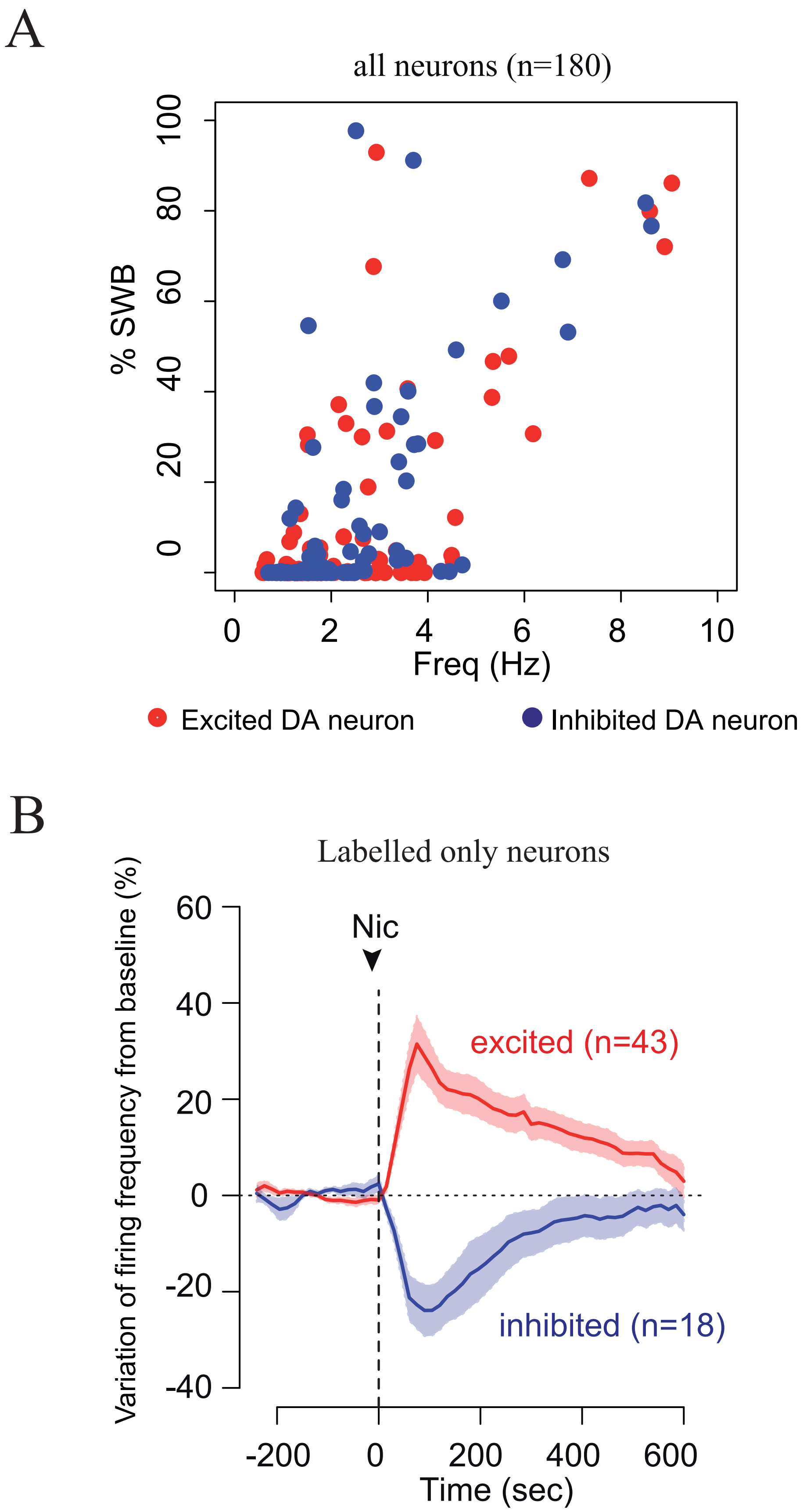

A
all neurons (n=180)
% SWB
0
20
40
60
80
100
0
2
4
6
8
10
Freq (Hz)
Excited DA neuron
Inhibited DA neuron
B
Labelled only neurons
Variation of firing frequency from baseline (%)
60
40
20
0
-20
-40
Nic
excited (n=43)
inhibited (n=18)
-200
0
200
400
600
Time (sec)

Figure S1 — **Spontaneous activity of excited versus inhibited cells**

A) Spontaneous activity of the excited (red, n=102) and inhibited (blue, n=78) neurons, represented by their mean firing frequency (Hz) against the percentage of spikes within a burst (%SWB). Spontaneous firing properties did not reveal any difference between spontaneous activity of excited and inhibited cells respectively: spontaneous firing rate: (2.6 ± 0.2 Hz and 2.5 ± 0.2 Hz, $p > 0.05$, asymptotic Wilcoxon rank sum test); spontaneous bursting: (10.7 ± 2.2 % and 13.5 ± 2.7 %, $p > 0.05$, asymptotic Wilcoxon rank sum test), except for the Coefficient of variation (0.46 ± 0.03 in excited cells, 0.56 ± 0.03 in inhibited cells, p =0.038, t-test). Finally, Action potential duration was analysed in 76 cells and was similar in the two populations (inhibited neurons: 2.4 ± 0.1 ms; excited neurons 2.5 ± 0.1 ms, $p > 0.05$, asymptotic Wilcoxon rank sum test, not shown). Overall, the spontaneous activity of a neuron doesn't predict or cannot be used to predict its response to nicotine. B) Mean responses of excited (red, n = 43) and inhibited (blue, n = 18) VTA DA neurons TH+ labelled cells. Data, expressed as variation of firing frequency from baseline, are presented as mean (thick curves) ± SEM (light curves). Arrowhead represents the time of nicotine injection.

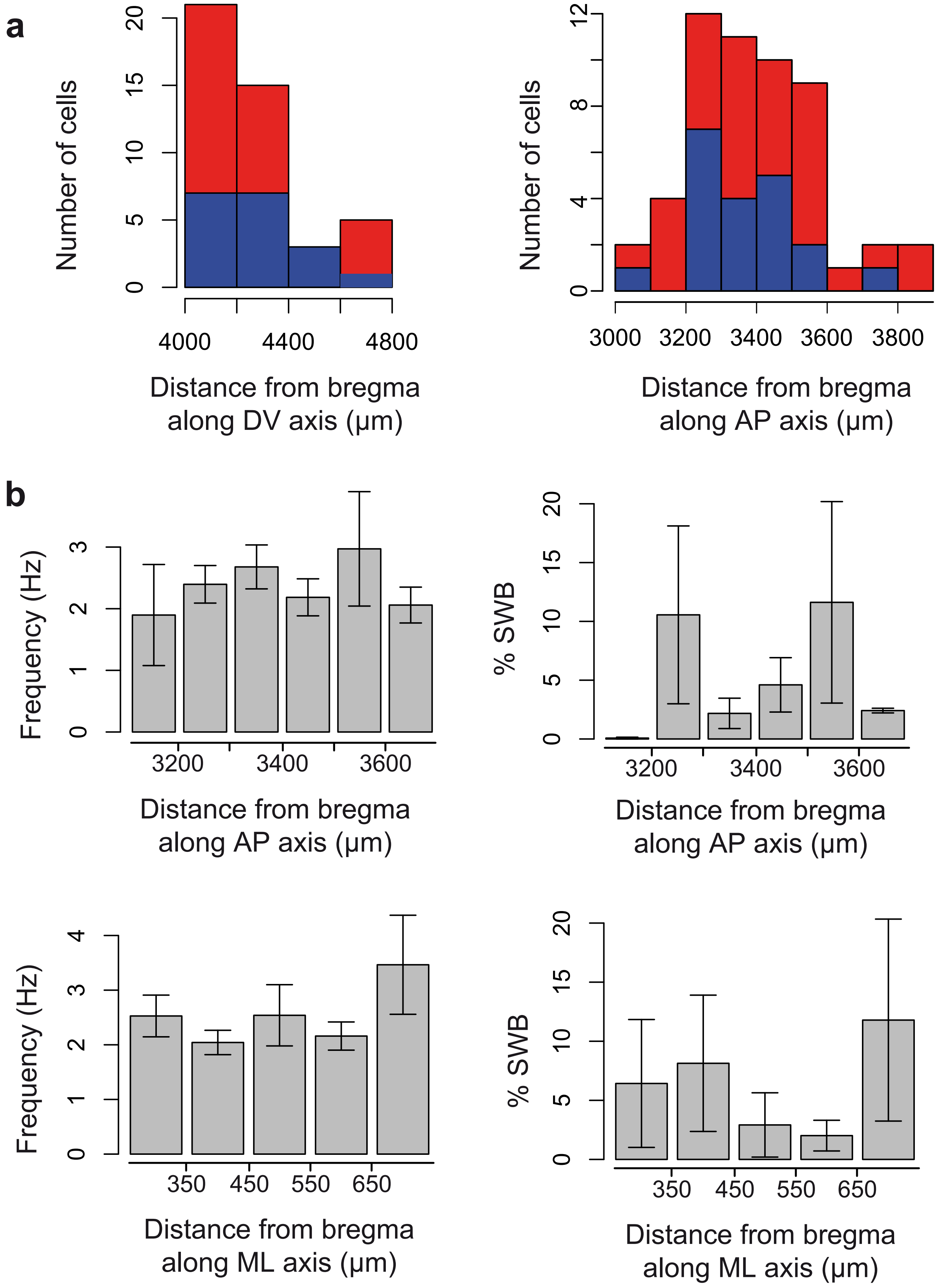
a
Number of cells
0
5
10
15
20
4000
4400
4800
Distance from bregma
along DV axis (μm)
Number of cells
0
4
8
12
3000
3200
3400
3600
3800
Distance from bregma
along AP axis (μm)
b
Frequency (Hz)
0
1
2
3
3200
3400
3600
Distance from bregma
along AP axis (μm)
% SWB
0
5
10
15
20
3200
3400
3600
Distance from bregma
along AP axis (μm)
Frequency (Hz)
0
1
2
3
4
350
450
550
650
Distance from bregma
along ML axis (μm)
% SWB
0
5
10
15
20
350
450
550
650
Distance from bregma
along ML axis (μm)

Figure S2 — **Localization and activity of DA neurons along the dorsoventral and anteroposterior axes**

**a** — Histogram of the anatomical distribution of all labelled excited and inhibited VTA DA neurons along dorso-ventral (left) and antero-posterior (right) axes. Inhibited and excited VTA DA neurons are not segregated along dorso-ventral (from the bregma, inhibited neurons located at 4223 ± 35 µm and excited neurons at 4201 ± 18 µm, $p > 0.05$, Wilcox test) and antero-posterior axes (from the bregma, inhibited neurons located at 3412 ± 44 µm and excited neurons at 3443 ± 28 µm, $p > 0.05$, Wilcox test).

**b** — Averaged firing frequency (left) and bursting activity (right) of all labeled neurons distributed according to their localizations along the antero-posterior (top) and medio-lateral (bottom) axes.

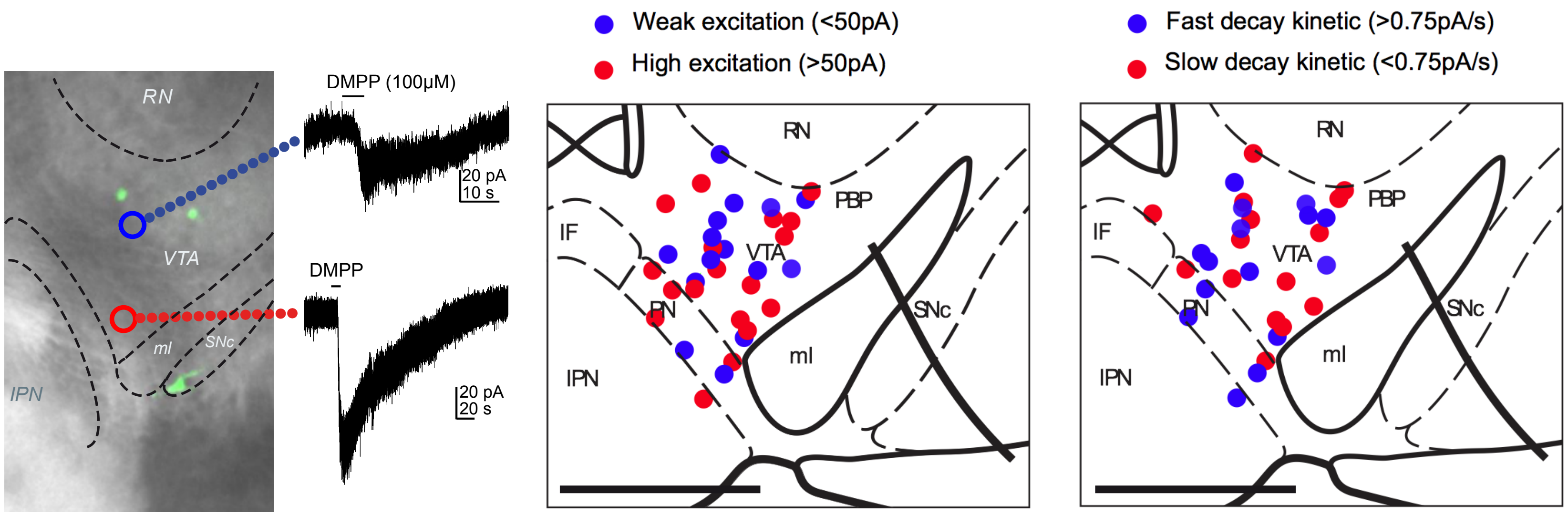

RN
VTA
IPN
ml
SNc
DMPP (100µM)
20 pA
10 s
DMPP
20 pA
20 s
Weak excitation (<50pA)
High excitation (>50pA)
RN
PBP
IF
VTA
PN
SNc
ml
IPN
Fast decay kinetic (>0.75pA/s)
Slow decay kinetic (<0.75pA/s)
RN
PBP
IF
VTA
PN
SNc
ml
IPN

Figure S3 — ***in vitro* electrophysiological analysis of VTA DA neurons in response to DMPP**

Electrophysiological recordings of nicotinic currents induced by local application of DMPP (1,1-dimethyl-4-phenylpiperazinium iodide, 100µM), a nicotinic agonist, in DA neurons throughout the VTA (nicotine is membrane permeant and is therefore very difficult to wash, contrarily to DMPP). Post-hoc labelling of recorded neurons show that nicotinic current amplitude does not depend on the localisation of the neuron in the VTA. We did not observe any differences in DA cells' nicotinic currents based on their anatomical position along the mediolateral axis (medial and lateral amplitude respectively: 88,1±17,5pA; 85,4±17,3pA p=0,8; medial and lateral decay kinetics respectively: 0,59±0,18pA/sec; 0,93±0,19pA/sec p=0,09 Mann-Whitney test). This indicates that the regional differences we observe could arise from network-dependent dynamics instead of reflecting differences based on nAChRs.

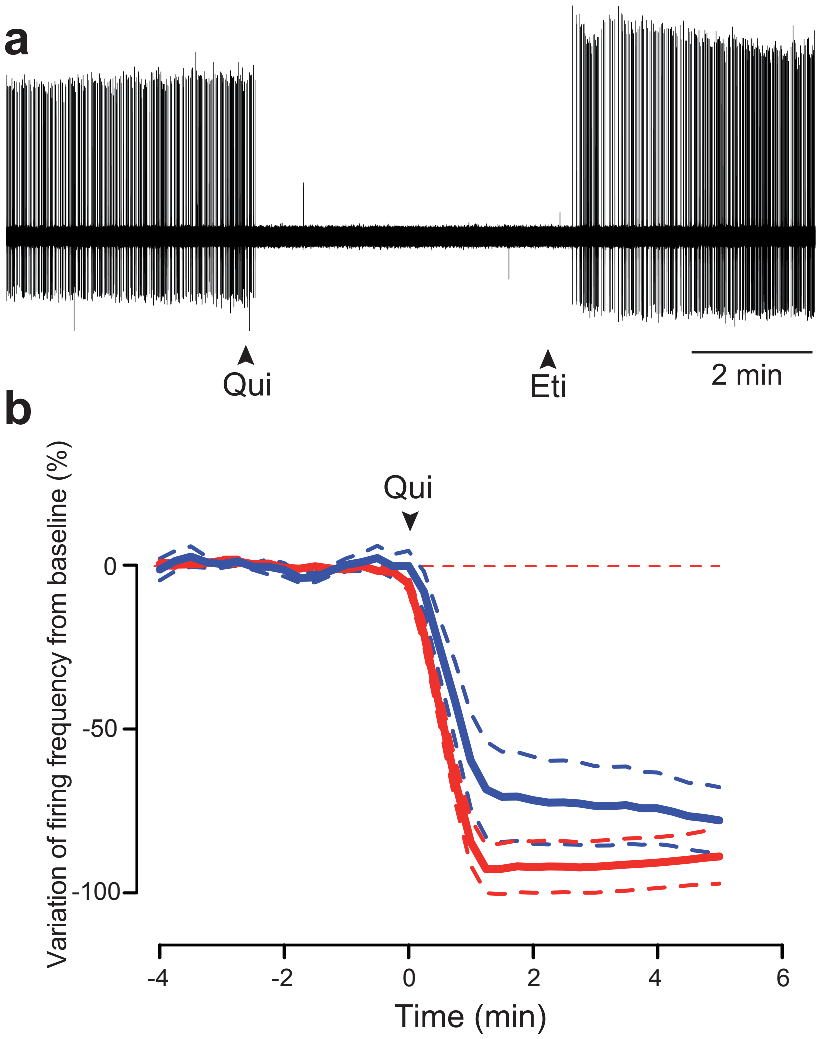
a
Qui
Eti
2 min
b
Variation of firing frequency from baseline (%)
Qui
0
-50
-100
-4
-2
0
2
4
6
Time (min)

Figure S4 — **Response of excited and inhibited VTA DA neurons to quinpirole injection.**

**a** — Raw extracellular recording of a single VTA DA neuron, displaying quinpirole (1mg/kg, Qui labelled arrowhead) and eticlopride (1mg/kg, Eti labelled arrowhead) intravenous injections.

**b** — Mean responses of excited (red, n=10) and inhibited (blue, n=10) VTA DA neurons. Data, expressed as variation of firing frequency from baseline, are presented as mean (thick curves) ± SEM (dotted curves). Red dotted line and arrowhead respectively represent the baseline and time of quinpirole injection.
